# Clinical Conditions Targeted by OnabotulinumtoxinA in Different Ways in Medicine

**DOI:** 10.3390/toxins16070309

**Published:** 2024-07-07

**Authors:** Dilara Onan, Fatemeh Farham, Paolo Martelletti

**Affiliations:** 1 Department of Physiotherapy and Rehabilitation, Faculty of Heath Sciences, Yozgat Bozok University, Yozgat 66000, Turkey; 2 Department of Headache, Iranian Centre of Neurological Research, Neuroscience Institute, Tehran University of Medical Sciences, Tehran 1417653761, Iran; farhamf3@mums.ac.ir; 3 School of Health, Unitelma Sapienza University of Rome, 00161 Rome, Italy; paolo.martelletti@unitelmasapienza.it

**Keywords:** OnabotulinumtoxinA, chronic migraine, spasticity, wrinkle, excessive sweating, CGRP, trigeminal neuralgia, hair loss

## Abstract

OnabotulinumtoxinA (BT-A) is used in different medical fields for its beneficial effects. BT-A, a toxin originally produced by the bacterium Clostridium botulinum, is widely known for its ability to temporarily paralyze muscles by blocking the release of acetylcholine, a neurotransmitter involved in muscle contraction. The literature continually reports new hypotheses regarding potential applications that do not consider blockade of acetylcholine release at the neuromuscular junction as a common pathway. In this opinion article, it is our aim to investigate the different pathway targets of BT-A in different medical applications. First of all, the acetylcholine effect of BT-A is used to reduce wrinkles for cosmetic purposes, in the treatment of urological problems, excessive sweating, temporomandibular joint disorders, obesity, migraine, spasticity in neurological diseases, and in various cases of muscle overactivity such as cervical dystonia, blepharospasm, and essential head tremor. In another potential pathway, glutamate A, CGRP, and substance P are targeted for pain inhibition with BT-A application in conditions such as migraine, trigeminal neuralgia, neuropathic pain, and myofascial pain syndrome. On the other hand, as a mechanism different from acetylcholine and pain mediators, BT-A is used in the treatment of hair loss by increasing oxygenation and targeting transforming growth factor-beta 1 cells. In addition, the effect of BT-A on the apoptosis of cancer cells is also known and is being developed. The benefits of BT-A applied in different doses to different regions for different medical purposes are shown in literature studies, and it is also emphasized in those studies that repeating the applications increases the benefits in the long term. The use of BT-A continues to expand as researchers discover new potential therapeutic uses for this versatile toxin.

## 1. Introduction

While the first mention of botulism appeared in the Middle Ages, interest in systematic studies increased greatly with food poisoning epidemics in the 18th and 19th centuries. Kerner’s clinical observations and experiments on botulinum toxin (BT) and the idea of its therapeutic use date back to the 1800s [1,2]. Symptoms in individuals have been reported after consuming unpreserved foods, and it has been stated that this condition is caused by bacteria [3]. Subsequent studies have shown that these anaerobic Clostridium botulinum bacteria produce a neurotoxin-active substance. Although neurotoxin A and B types are known to cause disease, they are used for their medical effects.

**Figure 1:**
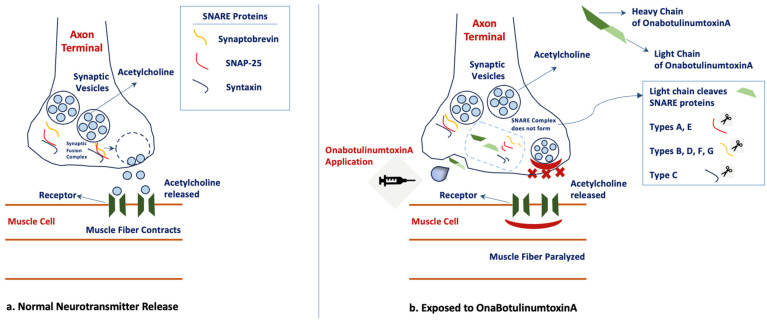
The mechanism of action of BT-A and acetylcholine.

It has been found that BT acts on motor and parasympathetic nerve endings, and the release of acetylcholine from presynaptic vesicles at the neuromuscular junction is inhibited by BT serotype A [3,4,5,6]. When BT-A is injected into the muscle, the C-terminus of the heavy chain binds to the receptor protein on the plasma membrane of the alpha motor neuron, and the toxin undergoes endocytosis. As the toxin changes in the environment, the disulfide bond decreases, causing the heavy chain and light chain to separate. The light chain passes from the endocytotic vesicle to the neuronal cytosol. In the cytosol, the light chain, acting as zinc endopeptidase, breaks down SNAP-25, the protein of the SNARE complex, which enables the association of vesicles containing acetylcholine (Ach) with the presynaptic membrane. Therefore, the Ach vesicle cannot bind to the presynaptic membrane, and ACh cannot be released into the neuromuscular junction [7,8]. This inhibition prevents nerve impulses and causes reversible muscle paralysis, which is dose-dependent [6]. The maximum effect of BT-A application occurs in the second week, and the clinical effects of BT-A generally last for 3–4 months [3,6,9]. With repeated applications, the effect of BT-A increases, and the duration of the effect is prolonged [5]. The mechanism of action of BT-A and acetylcholine is shown in Figure 1.

It is stated that the analgesic effect is due to the inhibition of peripheral and central sensitization of nociceptive fibers [10]. This effect occurs through the inhibition of pain mediators released from nerve endings, such as calcitonin gene-related peptide (CGRP), substance P, and glutamate [11,12,13,14,15,16]. At first, it was believed that BT-A reduced pain by inducing muscular relaxation while inhibiting the release of acetylcholine [17], but besides the Ach-releasing inhibition, there have been several preclinical studies showing that BT-A also inhibits the release of neurotransmitters that regulate pain and inflammation [13,18]. Particularly, BT-A inhibits the release of local nociceptive neuropeptides, such as glutamate, substance P, and CGRP [19,20,21]. Dorsal root ganglia neurons, mainly C fibers, produce substance P and CGRP, and BT-A inhibits the release of these neurotransmitters from primary sensory neurons [19]. Inhibiting the release of these neurotransmitters in areas where pain mediators are concentrated causes sensitivity and pain to decrease. In addition, delivery of the transient receptor potential vanilloid 1 (TRPV1) to neuron cell membranes could be decreased [22]. TRPV1 is an ionotropic receptor that responds to noxious stimuli, such as heat and pro-algesic substances [23]. TRPV1 has an important role in the processing of peripheral thermal and inflammatory pain. This mechanism could inhibit neurogenic inflammation, pain, and peripheral sensitization.

**Figure 2:**
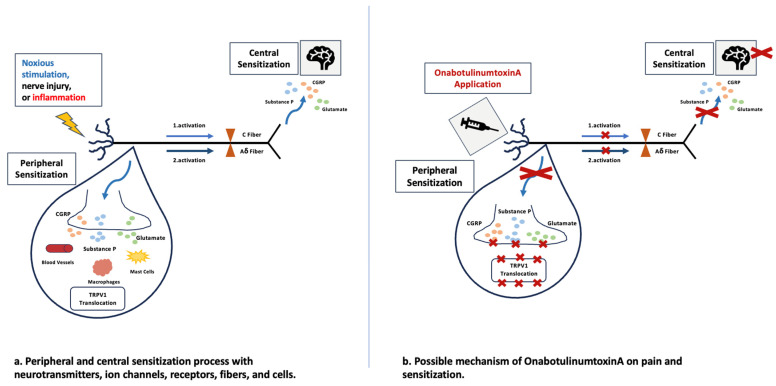
The mechanism of action of BT-A for pain inhibition.

Therefore, BT has an effect on muscle paralysis and pain inhibition through inhibition of acetylcholine and inhibition of sensitization. Although there are seven serotypes of the BT (A-G), serotypes A and B are used in medical practices. The mechanism of action of BT-A is shown in Figure 2.

BT-A is used for cosmetic purposes to reduce wrinkles, as well as to reduce spasticity in neurological conditions such as cerebral palsy, multiple sclerosis, and stroke, in the treatment of cervical dystonia and blepharospasm, in reducing the intensity and frequency of headache in chronic migraine, in essential head tremor, in hyperhidrosis and acral peeling skin syndrome, in urological problems such as incontinence, premature ejaculation, or overactive bladder, and in the treatment of diseases such as strabismus, temporomandibular joint disorders, hair loss, diabetic peripheral neuropathy, myofascial pain syndrome, and trigeminal neuralgia. Conditions approved by the U.S. Food and Drug Administration (FDA) are presented in Table 1. The effects discussed also apply to other botulinum toxin type A variants.

In this article, we aim to explain the use of BT-A in different clinical applications and how it can create a common effect pathway or a new different pathway hypothesis for its intended use.

## 2. Clinical Conditions in Which the Acetylcholine Mechanism Is Targeted with BT-A

### 2.1. Dermatological Use

BT-A is a frequently preferred adjunct application in the field of medical cosmetics due to its high effectiveness, high patient satisfaction, low side effects, and the absence of a surgical approach. Skin wrinkles become evident over time due to dermal atrophy, continuous muscle contractions, and a decrease in skin connective tissue and collagen [27]. This can cause a tired facial expression, such as pursing of the corners of the lips, lines on the forehead and around the eyes, and drooping eyelids. With the application of an appropriate dose of BT-A, these lines disappear. The mechanism of action of BT-A is by inhibiting acetylcholine and inhibiting muscle contraction, which is represented in Figure 1. After the contraindications are carefully evaluated, BT-A is applied to the targeted muscle areas of the face by considering the anatomical structure of the face [27]. The application dose of the toxin may vary depending on the targeted area, injection direction and depth, or gender [27]. Although the application targets muscle structure, on the other hand, there is an improvement in the skin and a decrease in the formation of oily skin. It is stated that locally, BT-A can cause a decrease in sebum by blocking the muscarinic acetylcholine receptor [28]. When the reported complications that may occur are examined, there may be pain or edema caused by injection at the application site, ecchymosis, asymmetries, ptosis, diplopia, lagophthalmus, difficulty breathing in nasal applications, difficulty in eating/drinking/chewing functions, or neck weakness. Experts state that all these effects may vary depending on dose adjustment, and careful monitoring is necessary. Although application doses may vary depending on the targeted location on the face [29], it is stated that BT-A applications are effective within 4-6 months, and as the duration of effect increases, the satisfaction of individuals increases [30,31].

### 2.2. Dystonias

- Cervical Dystonia: Cervical dystonia is a focal dystonia characterized by repetitive and involuntary contractions in the shoulder and neck muscles, accompanied by abnormal posture in the head, neck, and shoulders [32]. Patients’ complaints of tremor, pain, and disability negatively affect their quality of life, both functionally and psychologically. Pharmacological drugs are included in the treatment. Although it is stated that the healing effectiveness of physiotherapy and rehabilitation treatment increases when applied with BT-A and that cognitive behavioral therapies can also be recommended, the evidence strength is insufficient [33,34]. Surgical procedures can be performed when necessary in cases such as degenerative changes in the spine, myeloradiculopathy, or atlantoaxial instability [35]. In light of the literature, the first preferred method in the treatment of cervical dystonia is local BT-A application. BT-A acts by inhibiting the release of acetylcholine at the neuromuscular junction, blocking neuromuscular transmission. BT-A can be applied to the sternocleideomastoid, splenius capitis, levator scapula, semispinalis, trapezius, and scalene muscles, and depending on the clinical condition of the patient, the application of BT-A may involve several muscles or all the muscles just mentioned. The treatment effect starts within a week, and the duration of the effect can last up to 6 months [32]. Applications are made at at least 3-month intervals, keeping in mind that antibody formation occurs. Recommended doses may vary depending on the patient and the muscles to be applied; the range of doses for the injection can be a range of 198/200 to 360 units [36,37]. Evidence level A has been reported for BT-A in improving cervical dystonia, level A has been reported for reducing pain intensity, and level B has been reported for improving quality of life [38,39].
- Blepharospasm: Blepharospasm is a dystonia that occurs with spasms of the orbicularis oculi muscles, causing involuntary closure of the eyelid [40]. Periocular muscles may also be affected. Increased blinking and difficulty in involuntary eyelid closing and opening generally affect both eyes symmetrically. This difficulty in opening and closing the eyes may affect patients’ ability to perform their daily activities, and patients may have difficulty performing their duties during the day [40]. BT-A applied to the affected eye contour muscles reduces muscle contraction by blocking presynaptic acetylcholine release at the neuromuscular junction [41].

### 2.3. Neurological Diseases Accompanied by Spasticity

- Cerebral Palsy in Children: Cerebral palsy is a disease characterized by brain damage occurring in the early stages of development, where pyramidal, extrapyramidal dysfunction, and apraxic problems occur together [42]. In extrapyramidal dysfunctions, spasticity, dystonia, rigidity, spasm, or chorea findings may be observed. As the child grows, it is also possible to experience consequences such as contractures, joint problems, or growth retardation [42]. After the diagnosis is made in the treatment of cerebral palsy, physiotherapy and rehabilitation treatment take place throughout the process with the participation of the child and their family [43]. Considering growth and muscle joint development, BT-A application is also widely used in treatment to improve hyperactivity, especially in the lower extremity muscles [43,44]. The toxin blocks the release of acetylcholine at the neuromuscular junction, causing flaccid paralysis [43]. The effects of the BT-A application last for 3-6 months [45]. However, considering the child’s growth and development, good clinical observation is required so that the muscle relaxation effect of BT-A can proceed healthily with the child’s normal development [45]. With BT-A application, reduction in pain and spasticity [46,47], increase in gross motor function and joint range of motion, improvement in gait pattern, and increase in quality of life in children with cerebral palsy have been shown in literature studies for periods ranging from 12 weeks to 2 years [43,48,49]. Although the reported side effects such as pain, weakness, and fatigue after the application are mild, it is stated that recommending BT-A application together with physiotherapy, rehabilitation treatment, and orthoses can minimize possible surgical operations in the future [43].
- Upper Motor Neuron Diseases in Adults: Spasticity, which is combated in upper motor neuron lesion diseases such as multiple sclerosis, stroke, spinal cord injuries, and traumatic brain injuries, is among the most important causes of disability in adults. Again, problems such as joint deformities, contractures, and pain caused by spasticity affect the quality of life, emphasizing the need for the treatment process to be handled correctly [50]. BT-A has a level of evidence of A for focal spasticity in the upper and lower extremities [46,47,51]. BT-A, which has reversible effects, provides positive effects on individuals by improving walking patterns and mobility, increasing joint range of motion, reducing pain, and increasing the quality of life in daily living activities [52,53,54,55,56]. For example, spasticity, which is seen in almost 80% of patients with multiple sclerosis, may vary depending on the attack. In spinal cord injuries, spasticity is observed in the majority of patients until the first year after the injury. In traumatic brain injury, spasticity can develop very rapidly in the acute phase [50]. It is stated that BT-A treatment applied after the necessary evaluations in these diseases shows effects for 3–6 months, and the effectiveness of rehabilitation treatments may increase with the decrease in pain and spasticity [50]. In order to increase the duration of the effectiveness of BT-A and reduce the interruption of treatment, the time between injections should be carefully evaluated and not kept long [56]. Therefore, it is emphasized that BT-A treatment is a good aid because it is an important part of multimodal treatment for these diseases [50].

### 2.4. Essential Head Tremor

BT-A, which is also used in the treatment of essential head tremor, aims to reduce tremor with the effect of neuromuscular junction blockade. BT-A is applied to the splenius capitis and sternocleideomastoid muscles and has been shown in literature studies to be useful in reducing the severity of head tremor up to the 18th week [57,58]. Although the side effects of BT-A are minimal, temporary effects such as local pain at the injection site, neck weakness, muscle weakness, and dysphagia may be observed. However, it is emphasized that this requires dose adjustment and careful monitoring. On the other hand, it is noteworthy that studies on the effectiveness of BT-A in the treatment of essential head tremor are limited. It is anticipated that clearer results can be obtained with studies of strong methodological quality [57,58].

### 2.5. Urological Problems

- Incontinence and Related Problems: In cases of involuntary contraction or insufficient relaxation of the sphincter in urological disorders, BT-A is used by targeting the inhibitory mechanism of acetylcholine release at the neuromuscular junction in the striated muscle [59,60,61]. Normalization of continence, an increase in cystometric bladder capacity, an increase in maximum urine flow rate, a decrease in urinary incontinence, catheterization, improvements in mean detrusor pressure, and quality of life are achieved. In addition, these effects can last up to almost 12 months with repeated injections [62,63,64,65].
- Ejaculation Problems: BT-A is among the treatment methods for premature ejaculation problems. BT-A aims to inhibit the rhythmic contraction of the bullospongiosus and ischiocavernosus muscles by blocking presynaptic neuron activity and reducing norepinephrine flow [66]. It is aimed at increasing nitric oxide formation by blocking the release of acetylcholine from cholinergic neurons [67]. As a result, normal erection is achieved by relaxing the bullospongiosus and ischiocavernosus muscles [67]. Literature studies report that patients do not have prolonged intravaginal ejaculatory delay times or complications after BT-A application. In addition, the long duration of action and ease of application are stated as advantages of BT-A [68,69].

### 2.6. Strabismus

Strabismus, on the other hand, is a condition in which ocular alignment is deviated by the line of sight deviating by at least 1 prism diopter [70]. Therefore, the eyes are misaligned, with one eye looking fixedly while the other eye looks in another direction. Depth perception may be impaired, causing double vision or blurred vision. Activities in individuals’ daily lives and their quality of life may also be affected [70]. BT-A also allows the eyes to be aligned in a balanced, straight manner due to the effect of acetylcholine reducing muscle contraction [41].

### 2.7. Hyperhidrosis and Acral Peeling Skin Syndrome

Although sweating is a natural thermoregulation response, excessive and uncontrolled sweating can cause problems in individuals’ lives [71,72]. Although the pathophysiology of hyperhidrosis is not clearly understood, it is thought to be a problem in the autonomic nervous system that may cause overstimulation of the sweat glands [73]. Sweat glands are innervated by sympathetic cholinergic nerve fibers, and sweating occurs with the stimulation of sympathetic cholinergic fibers [73]. The body may respond with excessive sweating as a response to situations such as stress triggered by sympathetic cholinergic nerves [74]. There may be negative effects on individuals’ daily lives [74]. BT-A is used in the treatment of hyperhidrosis by inhibiting acetylcholine from overactivated cholinergic sympathetic nerve endings [74]. BT-A treatment has been reported to reduce excessive sweating in hyperhidrosis patients within 2 days to 1 week and last up to almost 12 months [74].

In acral peeling skin syndrome, sweat glands are targeted with the same mechanism for BT-A application. Acral peeling skin syndrome is an autosomal recessive disease that occurs as a result of a transglutaminase 5 gene mutation [75]. Although exfoliation is observed on the skin, lesions increase with moist and hot environments, increased sweating, or trauma. Acral peeling skin syndrome and BT-A studies are very limited in the literature. However, according to the results reported in the case reports, it has been reported that no significant skin peeling was observed after BT-A was applied to the areas of the hands and feet of patients with acral peeling skin syndrome aged 13, 23, and 51. However, since case studies have been reported, it has been observed that there is no common application protocol for BT-A [76,77].

### 2.8. Temporomandibular Joint Disorders

TMDs such as mandibular movement dysfunction and joint click usually present with pain, mandibular dysfunction, or joint sounds [78]. BT-A is used for the treatment of hyperactivity of the lateral pterygoid (LP) and other masticatory muscles after conservative treatments [79]. Toxin injections cure the hypertonicity of LP muscles and their consequent bruxism [80]. Also, injections in patients with articular displacement resulted in pain relief and a return to normal function. It is stated that the treatment is effective, and the effect continues for up to 6 weeks [81].

### 2.9. Obesity

Obesity is a major public health problem worldwide [82]. Obesity-related comorbidities like cardiovascular, metabolic, and oncologic issues are challenging for public health [83]. Changing dietary habits and lifestyle, increasing physical activity, pharmacologic intervention, and bariatric surgery for extreme obesity are the options for weight loss. This approach shows limited duration and effect. Surgical treatment [84] is the most effective method for weight loss, but it is an invasive procedure [85]. After the successful usage of intragastric BT-A in animal models [86], the effect of this drug has been assessed as an obesity treatment [87]. BT-A inhibits smooth and striated muscle contractions [88]. Intramuscular administration of BT-A could affect gastric motility [89], and its injection into the muscular layer of both the fundus and antrum can lead to a delay in gastric emptying and an earlier satiety, which causes weight loss due to low food intake. The effect could last from a period of 7 days to a maximum of 3–6 months [90].

## 3. Clinical Conditions in Which the Acetylcholine Mechanism, CGRP, Glutamate, and Substance P Are Targeted by BT-A

### 3.1. Chronic Migraine

In addition to pharmacological approaches in the treatment of chronic migraine, BT-A application is an application with proven effectiveness in prophylactic treatment. It is also stated that it prevents muscle paralysis with its dose-dependent and reversible effects, neurogenic inflammation, and peripheral and central sensitization through the inhibition of local nociceptive neuropeptides such as glutamate A, CGRP, and substance P [5]. An increase in the transient receptor potential vanilloid type-1 receptor (TRPV1), which stimulates the release of CGRP and substance P, is observed in chronic migraine patients. It is stated that BT-A can reduce TRPV1 [91,92,93]. Although injection-related headaches, neck pain, or muscle weakness may be seen as side effects, the beneficial effects of BT-A application continue from the 4th week to 1 year [5,94,95,96,97,98,99]. It provides improvements in headache intensity, headache days, headache frequency, neck pain intensity, disability and quality of life, and frequency of medication intake [95,98,99,100,101]. Its effectiveness as a prophylaxis for episodic migraine has not been clearly established. In prophylactic treatment of migraine, patients may choose to discontinue treatment due to the side effects of pharmacological treatments, and BT-A is therefore becoming attractive as an adjunct to the migraine treatment preferred by patients [5,94,95].

### 3.2. Cancer

- Cancer pain after radiation and after surgery: Two types of pain may affect cancer patients: neuropathic pain due to lesions or after surgery, and local muscle spasms, especially after radiotherapy [102]. The analgesic effect occurs through two different mechanisms. Inhibition of acetylcholine release at the neuromuscular junction is mainly responsible for relieving pain caused by muscle spasms. In the case of neuropathic pain, the analgesic effect is mainly due to the inhibition of pain neurotransmitters in the peripheral and central sensory systems [103,104,105]. In the studies, the duration of effect of BT-A injections to relieve pain was 3–6 months [106].
- Repair and healing of parotid glands damaged by cancer, radiation, or surgery: Botulinum toxin injection was used for the treatment of parotid gland damage such as gustatory hyperhidrosis (GH), sialorrhea, fistula, and sialocele formation, a safe and effective way to control sialorrhea and to heal the fistula [107,108]. Anecdotal observations have shown that 3 to 7 years of treatment are safe [107]. Further studies are needed to confirm these results, and long-term safety should be investigated in controlled, prospective clinical studies.
- Effect on cancer treatment: There is little literature demonstrating that adding BT-A to cancer cell cultures reduces cell growth, induces apoptosis, and slows mitotic activity in various cancer cell lines, including prostate, breast, colon, and pancreatic tumors [109,110,111,112,113].

## 4. Clinical Conditions in Which CGRP, Glutamate, and Substance P Are Targeted by BT-A

### 4.1. Trigeminal Neuralgia

Trigeminal neuralgia (TN) is a pain condition that can be extremely severe and debilitating. The first treatment strategy for patients with TN is medication, and if the patient does not respond to the drug, non-pharmacological options are available. The BT-A method is successful in the management of TN while having fewer side effects than other non-pharmacological strategies such as microvascular decompression [114]. BT-A injections are performed subdermally in the pain divisions and also submucosally [115]. Improvement in pain is seen over 10 days, even persisting for 6 months in some patients [116,117]. The mechanism is inhibition of CGRP, substance P, and glutamate release, as well as a reduction in inflammation and the deactivation of sodium channels [118,119]. It has been reported that facial asymmetry, bleeding, ecchymosis, or hematoma at the injection site may occur as side effects, but correct dosage and supervision are important [120].

### 4.2. Neuropathic Pain

Neuropathic pain is caused by an injury or disease of the somatosensory system [120]. The results from recent studies have suggested that BT-A has antinociceptive effects in neuropathic pain such as postherpetic neuralgia, diabetic neuropathy, post-traumatic neuralgia, and complex regional pain syndrome [20,121,122,123,124]. The antinociceptive effects last for up to 12 weeks [123]. The antinociceptive mechanism of BT-A in neuropathic pain includes a direct block of peripheral sensitization due to a decrease in peripheral SP, CGRP, glutamate, and TRPV1 receptor translocation and also indirectly reduces central sensitization while SP and CGRP secretion are blocked within the central nervous system [103].

### 4.3. Myofascial Pain Syndrome

Myofascial pain syndrome (MPS) is a condition in which treatment varies with pharmacological and physiotherapy approaches [125,126]. In cases where the disease is chronic and the treatment is ineffective, physicians may recommend BT-A treatment [125]. Literature studies apply BT-A as 5 units (u) to 50 u for MPS [125]. However, there is no consensus regarding the clinical success of BT-A in the treatment and recovery of MPS [125]. It is stated that although BT-A provides benefits in reducing pain intensity [127,128] and quality of life for up to 12 weeks [128], it causes limitations for healthcare professionals and patients regarding this treatment due to the high side effects and the expensiveness of the treatment [125,129].

## 5. Other Mechanisms

### Hair Loss

**Figure 3:**
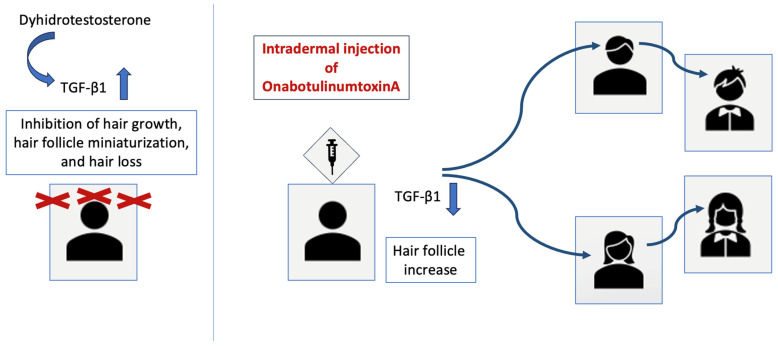
Potential mechanism of effect of BT-A in hair loss treatment.

The main reasons for hair loss are the narrowing of the vessels in the head area, the hair follicles not being fed adequately, and the dihydrotestosterone hormone binding to receptors in the hair follicles, affecting the hair growth cycle, thinning, and weakening the follicles [130]. If hair loss other than normal daily hair loss occurs on the scalp, treatment is required. The aim of the BT-A application is to regulate the circulation of blood vessels through muscle relaxation and increase oxygenation in the area. In addition, since dihydrotestosterone hormone causes the induction of transforming growth factor-beta 1 [TGF-β1] [130], intradermal injection with BT-A application aims to increase the hair follicles by decreasing the TGF-β1 activity in hair follicle cells [131] (Figure 3). Studies have shown that BT-A application in men and women shows an improvement in scalp oil secretion [132] and an increase in hair growth and density with applications up to 24 weeks, and it is reported that the application has safe therapeutic effects without side effects [131,133].

## 6. Conclusions

BT-A is used for muscle relaxation by targeting acetylcholine, pain control by reducing peripheral and central sensitivity by acting on CGRP, substance P, glutamate, and transforming growth factor-beta1, on hair follicles, and on the apoptosis of cancer cells. Therefore, BT-A is reported in new applications for medical conditions every day in the literature. This opinion article explains the mechanisms by which the BT-A treatment has been targeted and applied so far.

## Figures and Tables

**Table 1:** FDA-approved indications of BT-A [24,25,26].

Botulinum Toxin	FDA-Approved Indications
OnabotulinumtoxinA	Chronic migraine Overactive bladder Urinary incontinence Neurogenic detrusor overactivity Cervical dystonia Limb spasticity Axillary hyperhidrosis Strabismus Blepharospasm Hemifacial spasm Glabellar wrinkles (cosmetic)

FDA: Food and Drug Administration.

## Data Availability

Not applicable. No new data has been created.
